# 

**Published:** 1891-08

**Authors:** 


### HALL'S JOURNAL OF HEALTH

ESTABLISHED 1854

CONTENTS

Vol. 38 No. 8.

| | PAGE. |
|---|---|
| Sleep and Health | 169 |
| Cleanliness and Health | 171 |
| How Tobacco Smoking injures Yale Students | 171 |
| The Land of Pluck | 172 |
| The Baby | 173 |
| African Pygmies | 174 |
| Whitening the Hands and Face | 175 |
| Tan Spots | 176 |
| Warts | 177 |
| The Medicinal value of the Yellow Marigold | 178 |
| Extraordinary Case of Somnambulism | 178 |
| A Boy Mesmerist | 179 |
| Standards of Beauty | 180 |
| Sunlight | 180 |
| Spiritual Evidence | 181 |
| Thwarting the Divine Will | 181 |
| How to Keep the Baby Well | 182 |
| Petrified in a Pit | 183 |
| Children's Teeth | 184 |
| Eight Weeks Without Food | 185 |
| Miscellaneous | 188 |
| Driver Ants | 188 |
| Formation of Chalk | 189 |
| Literary | 190 |
| If We Could Know | 192 |

INDEX TO ADVERTISEMENTS OF HALF A PAGE AND OVER.

| | PAGE. |
|---|---|
| Horsford's Acid Phosphate | I |
| Lydia E. Pinkham Med. Co. | II |
| Erie Medical Co | IV |
| Ruckel & Hendel | V |
| Dr. J. C. Ayer & Co | V |
| Herba Vita | VIII |
| Hall's Journal Premiums | IX |
| Hall's Journal of Health | X |
| Royal Baking Powder | XI |
| I. S. Johnson & Co | XI |

